# 

**Published:** 1842-10

**Authors:** 


### CONTENTS

OF NO. IV.

---

### ORIGINAL COMMUNICATIONS.

### ESSAYS AND CASES.

Art. I.—Injuries of the Superior Extremities. By W. L. Sutton, M. D., Georgetown, Ky. 241

Alt. II.—Miscellaneous Cases. By Samuel Hogg, M. D., late of Tennessee. - - - - 252

### REVIEWS.

Art. III.—Researches in Embryology, in Three Series. By Martin Barry, M. D., F. R. S., Fellow of the Royal College of Physicians in Edinburgh. - 257

### SELECTIONS FROM AMERICAN AND FOREIGN JOURNALS.

Case of enormously enlarged Stomach. - - - 293
Professional Incomes in London. - - - 296
Bourgery on the Anatomy of the Spleen. - - 297
Ligature of the External Iliac Artery for Femoral Aneurism. 299
Counter-irritants. - - - - - - 305

Abdominal Tumors. - - - - - - 306
Use of Moxa in Rheumatism. - - - - 309
Velpeau on Cysts of the Eyelid. - - - 311
Fluor Spar. - - - - - - - 313
"Report on the Medical College" again. - - 313
Homœopathy. - - - - - - - 319

